# HIV antibody testing and its correlates among heterosexual attendees of sexually transmitted disease clinics in China

**DOI:** 10.1186/1471-2458-13-44

**Published:** 2013-01-17

**Authors:** Qiaoqin Ma, Xiaohong Pan, Gaofeng Cai, Jiezhe Yan, Masako Ono-Kihara, Masahiro Kihara

**Affiliations:** 1 Center for Disease Control and Prevention of Zhejiang Province, Hangzhou, 310051, People’s Republic of China; 2 Department of Global Health and Socio-epidemiology, Kyoto University School of Public Health, Kyoto, 606-8501, Japan

**Keywords:** HIV, HIV antibody testing, Sexually transmitted diseases, China

## Abstract

**Background:**

This study was conducted to determine the prevalence of HIV antibody testing and associated factors among heterosexual sexually transmitted disease (STD) clinic attendees in China.

**Methods:**

A self-administered questionnaire was administered among 823 attendees of 4 STD clinics of Zhejiang Province, China in October to December 2007. Psychosocial and behavioural factors associated with HIV antibody testing were identified in both genders using univariate and multivariate analyses.

**Results:**

Of all 823 STD clinic attendees, 9.3% of male and 18.0% of female attendees underwent HIV antibody testing in the most recent 6 months, and 60% of the participants had gotten no educational/behavioral intervention related to HIV prevention. The correlates for HIV antibody testing in the most recent 6 months as identified by multivariate analysis were ever condom use [odds ratio (OR), 10.37; 95% confidence interval (CI), 1.32–81.22]; ever anal/oral sex (OR, 3.13; 95% CI, 1.03–9.50) during their lifetime; having ever received three to seven types of behavioural interventions in the most recent 6 months (OR, 3.70; 95% CI, 1.32–10.36) among male subjects; and ever condom use (OR, 12.50; 95% CI, 2.20–71.01), STD history (OR, 3.86; 95% CI, 1.26–11.86) over their lifetime, or having ever received three to seven types of behavioural interventions in the most recent 6 months (OR, 8.68; 95% CI, 2.39–31.46) in female subjects. A lifetime experience of casual/commercial sex partners was strongly negatively associated with HIV testing in female subjects (OR, 0.08; 95% CI, 0.01–0.83).

**Conclusion:**

The low prevalence of HIV antibody testing and behavioural intervention among STD clinic attendees indicates a need for more targeted, intensive behavioural interventions to promote HIV antibody testing in this population.

## Background

The HIV epidemic in China continues to expand. It is driven by high-risk behaviour within particular sub-populations. The Ministry of Health, UNAIDS, and WHO assessed in 2009 that by the end of that year, approximately 740,000 Chinese people would be HIV-positive (range, 560,000–920,000) with an HIV prevalence of 0.057% (range, 0.042%–0.071%). Data from national sentinel surveillance and a survey in 61 cities showed that the HIV positive rates exceed 5% among men who have sex with men in some parts of China. Among affected individuals, heterosexual transmission accounts for 44.3% and homosexual transmission accounts for 14.7%. The proportion of sexual transmission is increasing annually; heterosexual transmission increased from 30.6% in 2006 to 47.1% in 2009, homosexual transmission increased from 2.5% in 2006 to 8.6% in 2009. Of the estimated 48,000 new HIV infections that took place in 2009, heterosexual transmission accounted for 42.2% and homosexual transmission accounted for 32.5%, sexual transmission is now the main mode of transmission of HIV [1]. Meanwhile, the prevalence of syphilis has increased rapidly in recent years, indicating the re-emergence of an epidemic. The reported national syphilis incidence increased from 7.39 per 100,000 in 2004 to 24.66 per 100,000 in 2009, which is an annual rate of increase of 26.6% [2]. The increasing prevalence of sexually transmitted diseases (STDs) has complicated the HIV epidemic in China.

Research conducted in developed and developing countries has shown that knowledge of one’s HIV status is the basis for accessing care and preventing further infection. HIV antibody testing could encourage safer behaviours of the tested individual to prevent contraction of HIV and other STDs. A positive test may prevent them from infecting others and encourage acquisition of early access to specific care, support, and anti-retroviral therapy [3-6].

In 2003, the Chinese government began to enforce the free voluntary counselling and testing (VCT) policy. In response to this, many VCT clinics, mainly at different levels of centres for disease prevention and control (CDC) have been established nationwide. In 2006, the Chinese government further required every county to establish at least 2 to 3 VCT clinics at CDC, and/or general hospitals, gynaecology and obstetrics hospitals (it is not compulsorily requested to establish VCT at STD clinic, which is usually affiliated to a general hospital or run independently)[7]. The number of VCT clinics climbed to approximately 8000 by 2010 [8]. However, by the end of 2009, the cumulative total of reported HIV-positive patients was 326,000. Reported cases and epidemic estimations show that about 55% of HIV-positive individuals have not been identified. The high proportion of HIV-positive individuals who do not know their status are particularly high-risk in terms of the potential spread of HIV infection, indicating the critical importance of promoting HIV testing in China.

In 2010, the State Council of China issued a document that stated the requirement to further strengthen HIV/AIDS prevention and control; one of its aims is expansion of the coverage of HIV/AIDS surveillance and testing services to the greatest extent possible [9]. To promote HIV testing, it is important to conduct surveys to elucidate the correlates of HIV testing among various populations. Some Chinese literature has documented that VCT could promote HIV testing among drug users [10], sex workers [11-13], men who have sex with men [14,15], premarital examinees [16], pregnant women [17-19], rural-to-urban migrants [6,20], and general adults [21,22]. Although the literature focuses on high-risk populations, including STD clinic attendees using VCT services [23,24], studies of HIV antibody testing and its correlates among STD clinic attendees are few, let alone among heterosexual STD clinic attendees. Given the high risk of sexual transmission of HIV among STD clinic attendees [25-28], it is crucial to understand the indicators that influence the performance of HIV testing to address the efficiency of identification of HIV cases among this population. Therefore, a cross-sectional survey of four STD clinics in Zhejiang Province was conducted.

## Methods

### Participants and data collection

The participants in the present study were derived from a cross-sectional survey conducted among attendees of four STD clinics in Zhejiang Province of Eastern China in October to December, 2007. In 2007, a total of 12 HIV surveillance sentinels at STD clinics performed surveillance on HIV prevalence and collected information on behaviours related to HIV transmission from April to June in Zhejiang Province. Of them, four clinics participated in this study; the remaining eight declined participation due to a lack of interest in this research after review of the study protocol. Analysis was performed to compare the HIV prevalence and the attendees’ demographic characteristics in terms of gender, age, marital status, and residence among the four STD clinics and the remaining eight non-participating clinics. The analysis revealed that significant difference was not shown for HIV prevalence (0.40% versus 0.23%) and these demographic characteristics were generally evenly distributed between the two groups. In principle, all >14-year-old sexually active attendees visiting these four STD clinics for diagnosis and treatment of STDs were included in the research. Those attendees who were not sexually active, not willing to participate in the research, had a language barrier, or visited clinics for general skin diseases were excluded from the research. During the study period, 1187 individuals (721 males, 466 females) visited the clinics for STD-related problems, and 935 (601 males, 334 females) agreed to participate in the research. The response rate was 83.4% for males, 71.7% for females, and 78.8% overall. Of the 935 respondents, 908 responded validly.

Of the 908 attendees, 16 of 586 males and 6 of 322 females had ever participated in sex with a same-sex partner and were not included in the analysis, resulting in a sample size of 886. Those attendees who completed the questions regarding HIV antibody testing in the most recent 6 months were included in the analysis; therefore, the final sample size of this study was 823.

The questionnaire was developed based on that used for HIV surveillance at the STD clinics at that time and a thorough review of the domestic and international literature, then modified according to repeated discussions among the research team and the doctors and nurses at the clinics studied. The final questionnaire comprised five sections that contained 7, 10, 21, 8, 5, questions, respectively. The questionnaires were self-administered and anonymous and were collected by doctors or nurses of the clinics during October and December, 2007.

### Ethical considerations

This research was ratified by Zhejiang Provincial Health Ministry. The institutional review board of Zhejiang Provincial Centre for Disease Prevention and Control reviewed the protocol and approved this research. All attendees of the four clinics who met the criteria for recruitment were advised of the study’s purpose and assured that their privacy and confidentiality would be strictly protected. Every attendee was invited to participate in the research, but the final decision was theirs.

### Measures

Those attendees who engaged in any oral, anal, or vaginal sex activities were defined as sexually active. HIV antibody testing during the most recent 6 months was used as a dependent variable in the analysis. The independent variables used included sociodemographic measures, those related to sexual behaviour and STD/HIV risk awareness, and scales of HIV-related knowledge and intervention that each participant received. For each scale, Cronbach’s alpha coefficients for internal consistency and the range of scores were computed; participants were categorised into three or four groups according to the ranges of scores based on the frequency distribution of each scale.

The HIV-related knowledge scale included four statements about whether the number of HIV cases reported in Zhejiang province has increased rapidly in recent years, whether HIV is spreading from high-risk populations to the general population through sexual intercourse, whether STDs make a person more vulnerable to HIV, and whether correct use of a condom can reduce the transmission of HIV. There were three possible responses to each of these four statements: ‘correct’, ‘incorrect’, and ‘unsure’. The scores for this scale ranged from 0 to 4, with 4 reflecting a high level of knowledge, 0 reflecting a low level of knowledge, and 1 to 3 reflecting an intermediate level of knowledge. The Cronbach’s alpha coefficient for this scale was 0.826.

The intervention scale reflected the educational/interventional services that an attendee received during the most recent 6 months, including seven statements regarding whether an attendee had ever received any pamphlets, lubricants, condoms, STD diagnoses or treatments, individual-to-individual counselling related to HIV/STD, group training for HIV/STD prevention, or services other than those listed. The possible responses of each statement were ‘yes’ or ‘no’. This scale had a Cronbach’s alpha coefficient of 0.710, and scores ranged from 0 to 7, with scores of 3 through 7 classified as high, 0 representing a person who received no interventional services, and 1 and 2 representing a person received one or two types of services, respectively. The seven statements of this scale were first analysed separately in bivariate analyses. Associations were found between HIV antibody testing and each above-mentioned service other than pamphlet and lubricant distributions.

### Statistical analysis

Data were analysed using SPSS for Windows (version 17.0; SPSS Inc., Chicago, IL). Frequency distributions of the independent variables and the prevalence of HIV antibody testing were determined by univariate analysis. Associations between the dependent variable and each independent variable were computed using an odds ratio (OR) with corresponding 95% confidence interval (95% CI) and a *p* value based on a chi-square test of proportions. Variables identified as significantly associated with HIV antibody testing in the bivariate analyses were then entered into a multivariate logistic regression model to determine the independent contribution of each factor to prediction of HIV antibody testing. A backward elimination procedure was adopted with a *p* value of > 0.10 as the removal criterion. Age, residence, income per month, marital status, and educational background were fixed in the model to control for possible confounding effects. A *p* value of < 0.05 was considered to indicate statistical significance in these analyses.

## Results

### Characteristics of study participants

Of the 823 attendees, 517 were male and 306 were female. A total of 34% of males and 55% of females were aged < 30 years old, 69% of males and 65% of females were married, and 76% of males and 66% of females were local residents (Table 1). Most males and females had a junior high school education and above and earned an income of > 1000 yuan/RMB per month.

**Table 1:** Socio-demographic characteristics of the participants

	**Male**	**Female**	**Total**
	**(n = 517)%**^**a**^	**(n = 306)%**^**a**^	**(n = 823)%**^**a**^
Age			
<30	33.5	55.2	41.6
30-39	33.7	27.5	31.3
≥40	32.9	17.3	27.1
Marriage			
Single	22.2	14.4	19.3
Cohabitation	7.5	18.3	11.5
Married	69.2	65.4	67.8
Residence			
Local resident	76.2	65.7	72.3
Out of the city	20.7	30.8	24.5
Education			
Illerate/Primary school	13.2	19.0	15.3
Junior high school	47.0	52.9	49.2
High school and over	38.7	27.8	34.6
Income			
<1000	14.7	29.4	20.2
1000-1999	41.0	37.3	39.6
≥2000	37.5	15.7	29.4

^a^The percentage of respondents may not add up to 100% due to missing data.

### Behavioural and psychosocial factors and performance of HIV antibody testing

Of all participants, 9.3% (48) of males and 18.0% (55) of females (total, 12.5% [103]) underwent HIV antibody testing in the most recent 6 months.

Among females, but not males, those who had two or more sexual partners during their lifetime were less likely to report HIV testing during the most recent 6 months (OR, 0.50) (Table 2). Participants were classified into two groups with respect to type of sexual partner: those who had only regular partners (regular partner-only group) and those who had ever had a casual or commercial partner (ever-casual/commercial partner group). Those who engaged in ever-casual/commercial partnership were less likely to have undergone HIV testing (OR, 0.31) among females, but this was not significant among males. Condom use was strongly associated with HIV testing; the OR was 17.63 and 36.00 for sometimes and always users among males, respectively, and 3.52 for sometimes users among females. Participants were categorised into three groups with respect to type of sex: those who conducted only vaginal sex, those who ever conducted anal sex, and those who ever conducted oral sex (excluding anal sex). Ever oral sex was associated with HIV antibody testing among males (OR, 2.98), but not females. HIV testing was associated with a participant’s history of STDs among females (OR, 2.89), but not among males; however, HIV testing was associated with unintended pregnancies among male’s partner (OR, 1.97), but not among female.

**Table 2:** Bivariate correlates of HIV testing in the recent 6 month with sexual behavioural and psychosocial factors

** Variable**	**Male (n = 517)**				**Female (n = 306)**			
	**n(%)**^**a**^	**HIV testing**	**Crude OR (95% CI)**^**b**^	***P *value**	**n(%)**^**a**^	**HIV testing**	**Crude OR (95% CI)**^**b**^	***P *value**
Partner number over life time								
1	98 (19.0)	12.2	1.00		162 (52.9)	20.4	1.00	
≥ 2	384 (74.3)	7.3	0.56 (0.28-1.15	0.117	133 (43.5)	11.3	0.50 (0.26-0.96)	0.038
Partner type over life time								
Regular only	170 (32.9)	10.0	1.00		232 (75.8)	20.3	1.00	
Ever casual/commercia	342 (66.2)	8.8	0.87 (0.46-1.62)	0.651	68 (22.2)	7.4	0.31 (0.12-0.82)	0.018
Condom use over lifetime								
Never	133 (25.7)	0.8	1.00	98 (25.7)	8.2	1.00		
Sometime	365 (70.6)	11.8	17.63 (2.40-129.33)	0.005	193 (70.6)	23.8	3.52 (1.59-7.80	0.002
Always	14 (2.7)	21.4	36.00 (3.45-375.70)	0.003	10 (2.7)	10.0	1.25 (0.14-11.16)	0.842
Type of sex over lifetime								
Only vaginal	465 (89.9)	8.0	1.00		284 (92.8)	18.0	1.00	
Ever anal	3 (0.6)	33.3	5.78 (0.51-65.29)	0.156	6 (2.0)	16.7	0.91 (0.11-7.99)	0.935
Ever oral	44 (8.5)	20.5	2.98 (1.33-6.66)	0.008	10 (3.3)	0.0	0.00	0.999
STD history over lifetime								
No	432 (78.1)	10.3	1.00		211 (69.0)	13.3	1.00	
Yes	75 (19.4)	22.5	1.60 (0.76-3.36	0.219	85 (27.8)	30.6	2.89 (1.57-5.30	0.001
Unintended pregnancy overlifetime								
No	403 (77.9)	7.9	1.00		175 (57.2)	15.4	1.00	
Yes	110 (21.3)	14.5	1.97 (1.04-3.75)	0.038	128 (41.8)	21.9	1.54 (0.85-2.76)	0.152
STD risk								
Impossible/unsure	175 (33.8)	12.6	1.00		99 (32.4)	18.2	1.00	
Possible	332 (64.2)	7.8	0.59 (0.32-1.08)	0.086	203 (66.3)	17.2	0.94 (0.50-1.76)	0.84
HIV risk								
Impossible/unsure	490 (94.8)	8.4	1.00		298 (97.4)	17.8	1.00	
Possible	26 (5.0)	26.9	4.04 (1.60-10.16)	0.003	7 (2.3)	14.3	0.77 (0.09-6.53	0.811
HIV related knowledge								
0	120 (23.2)	1.7	1		88 (28.8)	12.5	1	
1-3	197 (38.1)	9.1	5.93 (1.35-26.04)	0.018	137 (44.8)	13.9	1.13 (0.51-2.50)	0.768
4	188 (36.4)	14.4	9.90 (2.31-42.43	0.002	74 (24.2)	31.1	3.16 (1.42-7.03)	0.005
Awareness that every county has established VCT site								
Incorrect/unsure	388 (75.0)	6.4	1		210 (68.6)	12.9	1	
Correct	123 (23.8)	17.1	2.99 (1.61-5.56)	0.001	90 (29.4)	28.9	2.75 (1.50-5.06)	0.001

^a^Percentages may not add up up to 100 due to missing data for some items;

^b^OR, odds ratio; CI, confidence inerval.

Those who thought it was possible to be infected with STDs showed no association with HIV testing in both genders, but those who thought it was possible to be infected with HIV were more likely to undergo HIV testing among males (OR, 4.04). Awareness that every county has established a VCT clinic that is free of charge and anonymous was associated with HIV testing (OR, 2.99 and 2.75 for males and females, respectively). It seems that there was a trend in that those with a higher level of knowledge were more likely to undergo HIV testing among both males and females compared with those with a lower level of knowledge. The OR for attendees with knowledge scores of 1–3 and 4 was 5.93 and 9.90 for males and 1.13 and 3.16 for females, respectively, compared with the reference indicator score of 0.

### Behavioural intervention and performance of HIV antibody testing

Regarding the type of intervention received during the most recent 6 months, 60% of the participants received no educational/intervention; 15–24% received condoms, STD/HIV counselling, and STD checks and treatments; and 3–10% received lubricants, pamphlets, training for STD/HIV prevention, and others interventions not mentioned above (Table 3). Participants who responded that they received condoms, services related to STD/HIV counselling, training for STD/HIV prevention, STD checks and treatments, and other services during the most recent 6 months were markedly more likely to have undergone HIV antibody testing in the most recent 6 months than were those who responded otherwise (OR, 1.91–6.41 for males and 4.42–9.05 for females). Whether a participant received pamphlets and lubricants was unrelated to HIV antibody testing.

**Table 3:** Bivariate correlates of HIV testing in the recent 6 month with various intervention exposed

**Variable**	**Male (n = 517)**				**Female (n = 306)**			
	**n(%)**^**a**^	**HIV testing**	**Crude OR (95% CI)**^**b**^	***P *value**	**n(%)**^**a**^	**HIV testing**	**Crude OR (95% CI)**^**b**^	***P *value**
Intervention								
Condom								
No	390 (75.4)	7.4	1.00		236 (77.1)	11.9	1.00	
Yes	121 (23.4)	15.7	2.32 (1.25-4.31)	0.008	67 (21.9)	37.3	4.42 (2.35-8.33)	0.000
Lubricant								
No	484 (93.6)	9.5	1		277 (90.5)	17.3	1	
Yes	25 (4.8)	8.0	0.83 (0.19-3.63)	0.802	24 (7.8)	16.7	0.95 (0.31-2.92)	0.934
Pamphlet								
No	460 (89.0)	9.1	1		272 (88.9)	16.5	1	
Yes	49 (9.5)	12.2	1.39 (0.56-3.45)	0.480	28 (9.2)	28.6	2.02 (0.84-4.87)	0.118
STD/HIV counselling								
No	425 ()82.2	5.9	1		254 (83.0)	11.8	1	
Yes	83 (16.1)	27.7	6.13 (3.27-11.49)	0.000	46 (15.0)	47.8	6.84 (3.42-13.68)	0.000
Training for STD/HIV Prevention								
No	491 (95.0)	8.6	1		286 (93.5)	15.7	1	
Yes	17 (3.3)	29.4	4.45 (1.50-13.25)	0.000	15 (4.9)	46.7	4.69 (1.62-13.57)	0.000
STD check/treatment								
No	405 (78.3)	5.2	1		228 (74.5)	9.6	1	
Yes	104 (20.1)	26.0	6.41 (3.45-11.93)	0.000	73 (23.9)	41.1	6.53 (3.44-12.40)	0.000
Other								
No	483 (93.3)	9.1	1		284 (92.8)	14.8	1	
Yes	25 (5.2)	16.0	1.91 (0.63-5.80)	0.000	18 (5.9)	61.1	90.5 (3.32-24.68)	0.000
Intervention scale								
0	308 (59.6)	5.2	1		182 (59.5)	9.3	1	
1	80 (15.5)	8.8	1.75 (0.69-4.41)	0.235	44 (14.4)	11.4	1.24 (0.43-3.58)	0.685
2	57 (11.0)	15.8	3.42 (1.43-8.18)	0.006	34 (11.1)	23.5	2.99 (1.17-7.62)	0.022
3-7	60 (11.6)	25.0	6.08 (2.81-13.15)	0.000	36 (11.8)	55.6	12.13 (5.31-27.70)	0.000

^a^Percentages may not add up to 100 due to missing data;

^b^OR, odds ratio; CI, confidence interval.

Our findings showed that receipt of a high number of intervention services was a strong indicator for undergoing HIV testing among both males and females. There was a clear trend showing that the greater the types of services an individual received, the more likely they were to have undergone HIV antibody testing. Only 5.0% of males and 9.0% of females received HIV testing among those who received no services during the most recent 6 months, which increased to 30.4% and 71.4% among male and female participants, respectively, who received three to six types of services.

### Multivariate analysis

Table 4 shows the results of multiple logistic regression analyses. After age, marital status, residence, educational background, income per month had been controlled for; ever condom use (OR, 10.37), and ever anal/oral sex (OR, 3.13) during their lifetime, having ever received three to seven types of behavioural interventions (OR, 3.70) among males; and sex only with a regular partner (OR, 1), ever condom use (OR, 12.50), and STD history (OR, 3.86) over their lifetime, and having ever received behavioural intervention (OR, 8.68) among females remained significant correlates of HIV testing during the most recent 6 months. In addition, in the multiple logistic regression model the trend for STD clinic attendees who received a greater number of types of intervention services showed that they were more likely to have been HIV tested compared with those who received fewer types of interventions.

**Table 4:** Multivariate analyses predicting HIV testing in the recent 6 months

** Variable**	**Male**		**Female**	
	**Adjusted OR (95% CI)**^**a**^	***P*-value**	**Adjusted OR (95% CI)**^**a**^	***P*-value**
Partner type over lifetime				
Regular only			1.00	
Ever casual/commercial			0.08 (0.01-0.83)	0.034
Condom use over lifetime				
Never	1.00		1.00	
Ever	10.37 (1.32-81.22)	0.026	12.50 (2.20-71.01)	0.004
Awareness that every county has established VCT site				
Incorrect/unsure	1.00			
Correct	2.07 (0.93-4.59)	0.075		
Type of sex over lifetime				
Only vaginal	1.00			
Ever anal or oral	3.13 (1.03-9.50)	0.044		
STD history over lifetime				
No			1.00	
Yes			3.86 (1.26-11.86)	0.018
Behavioural intervention				
0	1.00		1.00	
1	1.02 (0.31-3.38)	0.974	0.69 (0.14-3.40)	0.648
2	2.52 (0.93-6.84))	0.069	3.34 (0.85-13.22	0.085
3-7	3.70 (1.32-10.36)	0.013	8.68 (2.39-31.46	0.001

^a^OR, odds ration; CI, confidence interval.

Given the coexistent relationship between the numbers of casual/commercial partnerships and sexual partners, those who ever engaged in casual/commercial partnerships are probably more likely to have more than one sexual partner than those with regular partners. Therefore, we excluded the partner type over lifetime variable from the model, and found that those females with more than two sexual partners were less likely to have undergone HIV testing (OR, 0.27; 95% CI, 0.09–0.83), which corroborated the results of the bivariate analysis.

## Discussion

This is the first study in China specifically examining actual HIV testing correlates among heterosexual STD clinic attendees. Therefore, this study not only augments the limited data available on HIV testing among STD clinic attendees in China, but, more importantly, provides information valuable for development of more effective HIV testing services for this population.

Our data suggest that the prevalence of having undergone HIV antibody testing in the previous 6 months among STD clinic attendees was 9.3% for males and 18.0% for females, indicating that a high proportion of the STD clinic attendees had not undergone HIV antibody testing. The facts that the rate of always condom use was < 5% and multiple sexual partnerships and commercial/casual sex were quite prevalent among our participants suggests the importance of promotion of safer behaviours among this group. Such health behaviour promotion should include HIV testing because there is a concern that such a low testing rate may lead to a possible delay in the diagnosis of HIV/AIDS in this high-risk population.

HIV testing services are widely available in China, especially in economically developed areas such as Zhejiang Province, where all local CDCs and some hospitals offer free, anonymous, voluntary HIV counselling and testing services. In addition, the majority of hospitals in every county offer HIV testing if a doctor or patient believes it to be necessary. Considering the fact that free anti-retroviral therapy services were already available for all AIDS patients in China at the time of this study, the introduction of anti-retroviral therapy may dramatically drive people to get HIV testing, the low prevalence of HIV antibody testing in this population is surprising. This may be be related to the inadequacy of relevant HIV prevention programs. Our findings showed that ~60% of attendees had received no intervention services during the most recent 6 months. This may be related to inadequate risk assessment and mobilisation of doctors at hospitals and clinics, which was reported to be a predictor for undergoing HIV testing services [29-32]. However, many medical providers are reluctant to discuss HIV risk behaviour with a patient, which has already been reported in the US [33-35]. This may be related to the perception of a low risk of HIV infection. The majority of our participants believed that it was impossible for them to contract HIV. This may be related to fear of stigmatisation and a positive result [36], therefore, they refrained from testing. This may be possibly because of a low awareness of the VCT services that have been established in every county. Our results showed that awareness of VCT services is associated with HIV testing, but this factor was present in < 30% of both genders. Multiple efforts, therefore, should be made to remove the psychosocial barriers that prevent HIV testing, and counselling services and intervention programs should be provided for this risk group. STD clinic doctors’ recommendations and mobilisation may play an important role in this regard.

The present study did not replicate a previous finding that the self-perceived chance of HIV infection and knowledge variables were significantly associated with the incidence of HIV testing [29,37]. Instead, we found that STD clinic attendees who had sexual intercourse with commercial or casual sexual partners and females who had multiple lifetime sex partners were more likely to have been HIV tested. In addition, lifetime ever condom use was the strongest correlate of HIV testing among both males and females; this association has also been reported elsewhere [38]. These findings suggest that participants’ decisions regarding taking an HIV test are more likely to be based on personal sexual behaviours than estimation of personal risk or knowledge.

Reports from other countries regarding the relationship between HIV testing and the diagnosis of an STD have been inconsistent [37,38]. Females who had ever been tested were more likely to report an STD history in this study. This may be because some Chinese women who had suffered psychological trauma due to an STD or experience of STD diagnosis and treatment may be driven to, or subsequently adopt safer behaviours, including HIV testing, reinforcing the hypothesis that information on prevention through counselling, exam and treatment of gynaecological diseases can assist substantial behavioural modifications, allowing for safer sexual practices [39].

We found that heterosexual male attendees who had ever engaged in oral sex were more likely to have been tested. Although not shown, the data revealed that male participants who had ever practiced both oral and vaginal sex were more likely to have had multiple sexual partnerships and an STD compared with those who participated only in vaginal sex. However, they were more likely to believe that it was possible to contract HIV; unfortunately, their condom-use rate was not increased. Males who practiced oral sex were more sexually active and practiced higher-risk behaviours; therefore, they may be more aware of their risk and more likely to undergo HIV testing. In this study, 8.5% of heterosexual men had performed oral sex; the HIV and STD risk for this population should therefore be noted.

Reports from other countries indicate that heterosexual anal sex is prevalent among STD clinic patients, with more than one in five patients reporting anal sex in the previous 3 months and 39% reporting anal intercourse in the past year [40,41]. Although our research reported a much lower lifetime anal sex rate (0.6% for males and 2.0% females), although penile–anal intercourse is more efficient at transmitting HIV and STDs than is vaginal intercourse [42,43], anal sex was not associated with HIV testing. Because the number of heterosexual individuals engaging in anal sex practices in China may be increasing, which has also been reported for the US [44], future HIV prevention interventions for heterosexual men in China must attach importance to the risk of HIV posed by anal sex.

One of the most important findings of this study was the dosage-response relationship between type of intervention and HIV testing. A clear trend was shown in that the greater the number of types of intervention the participants received, the more likely they were to undergo HIV testing, implying that intervention programs must implement packages comprising various components to encourage STD clinic attendees to undergo HIV testing.

Our study has some limitations. First, the participants may differ from those who chose not to participate, but the direction of this bias is unknown. Second, HIV testing information was based on self-reports. The results may have been affected by the respondents’ concern about social desirability and whether they felt comfortable reporting such behaviours in STD clinics. However, HIV testing recall error might not have been evident because the time of recall was limited to 6 months. HIV testing is a stressful experience, and the respondents’ HIV status was not requested. Furthermore, some participants may have felt uncomfortable responding to the sexual behaviour questions; this may have led to underreporting bias. Third, we did not inquire whether HIV tests were initiated by the test-seeker or the provider; this should be taken into consideration in analyses of the incidence of HIV testing and its correlates. Fourth, the cross-sectional nature of this study limits the drawing of causal inferences between HIV testing and its correlates. The association of HIV testing with behavioural intervention might be due to that some participants may get HIV testing and behavioural intervention at VCT at the same time, however, we believe that the proportion for our participant to get HIV testing at VCT is low as we limited HIV testing to the previous 6 months, and the major concern for STD clinic attendee is STD, not HIV, which is reported in our study that HIV risk awareness is 5.0% and 14.3% for male and female, respectively.

## Conclusion

Our findings have important implications for development of intervention programs targeting STD clinic attendees in China. First, a high level of multiple sexual partnerships and low level of condom use was found, putting STD clinic attendees at risk of HIV infection and emphasising the importance of encouraging this population to undergo HIV testing. Second, more intensive and multiple behavioural interventions that promote HIV testing should be conducted within this population. Third, to make HIV testing services more available and accessible to STD patients, it may be helpful to establish VCT service in every STD clinic to not only provides STD diagnosis and treatment, but also counselling and testing. Finally, intervention programs intended to promote HIV testing among this population must be scientifically designed and take sexual behaviours and gender differences into account.

## Competing interests

The authors declare that there are no competing interests for this manuscript.

## Authors’ contributions

All authors contributed to the design of this research. MQ performed the statistical analysis and drafted the manuscript; PX coordinated the study in field; PX, CG helped analyze the data; YJ played a major role in the field survey. MOK and MK supervised statistical analysis and made critical comments on the manuscript. All the authors read and approved the contents of the manuscript.

## Pre-publication history

The pre-publication history for this paper can be accessed here:

http://www.biomedcentral.com/1471-2458/13/44/prepub

## References

[B1] State Council AIDS Working Committee Office UN Theme Group on AIDS in China A joint assessment of HIV/AIDS prevention, treatment, and care in China (2009) 2009 Beijing

[B2] Gong XD STD epidemiology and control Bulletin for STI prevention and control 2011 25 9 13

[B3] Kawichai S Beyrer C Khamboonruang C Celentano DD Natpratan C Rungruengthanakit K Nelson KE HIV incidence and risk behaviors after voluntary HIV counseling and testing (VCT) among adults aged 19–35 years living in peri-urban communities around Chiang Mai city in northern Thailand, 1999 AIDS Care 2004 16 21 35 10.1080/09540120310001633949 14660141

[B4] Weinhardt LS Carey MP Johnson BT Bickham NL Effects of HIV counseling and testing on sexual risk behavior: a meta-analytic review of published research, 1985–1997 Am J Public Health 1999 89 1397 1405 10.2105/AJPH.89.9.1397 10474559 PMC1508752

[B5] Voluntary HIV-1 Counseling and Testing Efficacy Study Group Efficacy of voluntary HIV-1 counseling and testing in individuals and couples in Kenya, Tanzania, and Trinidad: A randomized trial Lancet 2000 356 103 112 10963246

[B6] He N Zhang J Yao J Tian X Zhao G Jiang Q Detels R Knowledge, attitudes, and practices of voluntary HIV counseling and testing among rural migrants in Shanghai, China AIDS Educ Prev 2009 21 570 581 10.1521/aeap.2009.21.6.570 20030500 PMC2903536

[B7] State Council of China The five-year action plan for HIV/AIDS prevention and control (2006–2010) in China 2006 Beijing

[B8] Chinese Center for Disease Prevention and Control The data for HIV/STD prevention and control in China (first Quarter, 2010) 2010 Beijing

[B9] State Council of China Notice on the requirement of further strengthening HIV/AIDS prevention and control 2010 Beijing

[B10] Chen HT Liang S Liao Q Wang S Schumacher JE Creger TN Wilson CM Dong B Vermund SH HIV voluntary counseling and testing among injection drug users in south China: a study of a nongovernment organization based program AIDS Behav 2007 11 778 788 10.1007/s10461-007-9215-x 17347877

[B11] Li X Wang B Fang X Zhao R Stanton B Hong Y Dong B Liu W Zhou Y Liang S Yang H Short-term effect of a cultural adaptation of voluntary counseling and testing among female sex workers in China: a quasi-experimental trial AIDS Educ Prev 2006 18 406 419 10.1521/aeap.2006.18.5.406 17067252 PMC1933388

[B12] Sun L Huang C Lin Y Zhuang H Wang Y Fang L Li L Hou H Zhang Y Impact factors related to HIV voluntary counseling and testing Chin J AIDS STD 2007 13 357 359

[B13] Li L Wang J Lei J Wang T Lai X Liang J Luo X Case–control study on influence factors of HIV Voluntary Counseling and testing among female sex workers Occup and Health 2008 24 2683 2684

[B14] Wang Y Zhang H Zhang G Yang H Zheng Y Fan J AIDS Control Demand and Influence Factors of Biologic Monitoring Among MSM J Prev Med Inf 2008 24 161 165

[B15] Guo J Wang Y Ma J Analysis on influencing factors of HIV test among male homosexuality under structural equation Model J Prev Med Inf 2008 24 895 897

[B16] Wu Z Rou K Xu C Lou W Detels R Acceptability of HIV/AIDS counseling and testing among premarital couples in China AIDS Educ Prev 2005 17 12 21 10.1521/aeap.17.1.12.58686 15843107

[B17] Khoshnood K Wilson KS Filardo G Liu Z Keung NH Wu Z Assessing the efficacy of a voluntary HIV counseling and testing intervention for pregnant women and male partners in Urumqi City, China AIDS Behav 2006 10 671 681 10.1007/s10461-006-9092-8 16897353

[B18] Fang L Wang L Wang Q Use of HIV testing services among pregnant women and its influencing factor Chin J Public Health 2008 24 393 394

[B19] Fan A Guo G Li Z Li Y A study on HIV Counseling and testing among pregnant women and its correlates in Yunnan rural area China women children care 2007 22 2464 2467

[B20] Li X Fang X Lin D Mao R Wang J Cottrell L Harris C Stanton B HIV/STD risk behaviors and perceptions among rural-to-urban migrants in China AIDS Educ Prev 2004 16 538 556 10.1521/aeap.16.6.538.53787 15585430 PMC1791014

[B21] Ma W Detels R Feng Y Wu Z Shen L Li Y Li Z Chen F Wang A Liu T Acceptance of and barriers to voluntary HIV counseling and testing among adults in Guizhou province, China AIDS 2007 21 Suppl 8 S129 S135 10.1097/01.aids.0000304708.64294.3f 18172381 PMC2903547

[B22] Ma W Wu Z Qin Y Detels R Shen L Li Y Liu T Chen F Comparison of voluntary counseling and testing uptake between a China CARES county and a county not designated for the China CARES Program AIDS Patient Care STDS 2008 22 521 533 10.1089/apc.2007.0226 18462075 PMC2928493

[B23] Zou Y Pan X Yang J Xu Y Cen G Guo Z Study on the influence factors of HIV voluntary counseling and testing among commercial sex workers, drug users, and STD Clinic Attendees Chin Prev Med 2008 9 491 495

[B24] Yan H Ding P Chen G Xu J Huan X Yang H A study on factors influencing acceptability of HIV voluntary counseling and testing among HIV/AI DS related population at high risk Acta of Nanjing Med Univ (Natural Science) 2008 28 909 913

[B25] Choi KH Zheng X Qu S Yiee K Mandel J HIV risk among patients attending sexually transmitted disease clinics in China AIDS Behav 2000 4 111 119 10.1023/A:1009549110508

[B26] Wong SP Yin YP Gao X Wei WH Shi MQ Huang PY Wang H Chen Q Liu M Tucker JD Chen XS Cohen MS Risk of syphilis in STI clinic patients: a cross-sectional study of 11,500 cases in Guangxi, China Sex Transm Infect 2007 83 351 356 10.1136/sti.2007.025015 17591664 PMC2659023

[B27] Ma Q Han H Wu F Wang X Jin W Pan X Yang J Cai G Sexual behaviors among patients attending sexually transmitted disease clinics in Zhejiang Province Chin J Nat Med 2009 11 406 410

[B28] Chen XS Yin YP Tucker JD Gao X Cheng F Wang TF Wang HC Huang PY Cohen MS Detection of Acute and Established HIV Infections in Sexually Transmitted Disease Clinics in Guangxi, China: Implications for Screening and Prevention of HIV Infection J Infect Dis 2007 196 1654 1661 10.1086/522008 18008249

[B29] Zhang W Nie S Shi W Shi P Li G Zhu L Xu Y Yang T Study on factor influence on utilization of the voluntary HIV counseling and testing among population at high risk Modern Prev Med 2010 37 1405 1407 1410

[B30] Sawleshwarkar S Harrison C Britt H Mindel A Determinants of HIV testing Sex Transm Infect 2011 87 426 432 10.1136/sti.2011.049601 21685190

[B31] Mahoney MR Fogler J Weber S Goldschmidt RH Applying HIV Testing Guidelines in Clinical Practice Am Fam Physician 2009 80 1441 1444 20000306

[B32] Bokhour BG Solomon JL Knapp H Asch SM Gifford AL Barriers and Facilitators to Routine HIV Testing in VA Primary Care J Gen Intern Med 2009 24 1109 1114 10.1007/s11606-009-1078-6 19690923 PMC2762506

[B33] Mahajan AP Stemple L Shapiro MF King JB Cunningham WE Consistency of state statutes with the centers for disease control and prevention HIV testing recommendations for health care settings Ann Intern Med 2009 150 263 269 19221378 10.7326/0003-4819-150-4-200902170-00007 PMC2874823

[B34] Beckwith CG Flanigan TP del Rio C Simmons E Wing EJ Carpenter CC Bartlett JG It is time to implement routine, not risk based, HIV testing Clin Infect Dis 2005 40 1037 1040 10.1086/428620 15824997

[B35] Burke RC Sepkowitz KA Bernstein KT Karpati AM Myers JE Tsoi BW Begier EM Why don’t physicians test for HIV? A review of the US literature AIDS 2007 21 1617 1624 10.1097/QAD.0b013e32823f91ff 17630557

[B36] Worthington C Myers T Factors underlying anxiety in HIV testing: Risk perceptions, stigma, and the patient-provider power dynamic Qual Health Res 2003 13 636 655 10.1177/1049732303013005004 12756685

[B37] McGarrigle CA Mercer CH Fenton KA Copas AJ Wellings K Erens B Johnson AM The relationship between HIV testing and risk behaviour in Britain: National Survey of Sexual Attitudes and Lifestyles 2000 AIDS 2005 19 77 84 10.1097/00002030-200501030-00009 15627036

[B38] Scott-Sheldon LA Carey MP Carey KB Cain D Vermaak R Mthembu J Harel O Simbayi LC Kalichman SC Impact of HIV testing on sexual health communication in South Africa Sex Transm Infect 2011 87 242 247 10.1136/sti.2010.045732 21164149 PMC3722047

[B39] Ribeiro Filho AD Giraldo PC Silva MJ Amaral RL Eleutério Junior J Gonçalves AK Behavioral and biological risks of women seeking HIV test in an anonymous testing center Braz J Infect Dis 2011 15 360 364 10.1016/S1413-8670(11)70205-7 21861007

[B40] Tian LH Peterman TA Tao G Brooks LC Metcalf C Malotte CK Paul SM Douglas JM Jr RESPECT-2 Study Group Heterosexual anal sex activity in the year after an STD clinic visit Sex Transm Dis 2008 35 905 909 10.1097/OLQ.0b013e318181294b 18685549

[B41] Leichliter JS Heterosexual anal sex: part of an expanding sexual repertoire? Sex Transm Dis 2008 35 910 911 10.1097/OLQ.0b013e31818af12f 18813143

[B42] Boily MC Baggaley RF Wang L Masse B White RG Hayes RJ Alary M Heterosexual risk of HIV-1 infection per sexual act: systematic review and meta-analysis of observational studies Lancet Infect Dis 2009 9 118 129 10.1016/S1473-3099(09)70021-0 19179227 PMC4467783

[B43] Kalichman SC Simbayi LC Cain D Heterosexual anal intercourse among community and clinical settings in Cape Town, South Africa Sex Transm Infect 2009 85 411 415 10.1136/sti.2008.035287 19429569 PMC3017216

[B44] Misegades L Page-Shafer K Halperin D McFarland W YWS Study Investigators Group Anal intercourse among young low-income women in California: an overlooked risk factor for HIV? AIDS 2001 15 534 535 10.1097/00002030-200103090-00017 11242155

